# The Trend of Malaria Cases, Positivity Rate, and Determinant Factors in the Amhara Regional State, Ethiopia: A Mixed Method

**DOI:** 10.1155/2021/2131720

**Published:** 2021-12-20

**Authors:** Chalachew Yenew, Sileshi Mulatu, Asaye Alamneh

**Affiliations:** ^1^Social and Population Health (SPH) Unit, Debre Tabor University, Debre Tabor, Ethiopia; ^2^Pediatrics and Child Health Nursing, Bahir Dar University, Bahir Dar, Ethiopia

## Abstract

**Objectives:**

The objectives of this study were to evaluate the trend of malaria cases and test positivity rate and explore determinant factors in the Amhara Regional State, Ethiopia.

**Methods:**

A mixed study design (retrospective record data review and case study) was employed among 67 malaria officers from all zones in the region by using proportional allocation and the 1995 to 2020 malaria document review. 1995 to 2020 trend analysis was conducted using RStudio-1.2.5033. Vignette Focus Group Discussions (FGDs) were used to dig the possible factors for malaria case buildup using the purposive sampling technique, and a qualitative content analysis was used.

**Results:**

The overall mean test positivity rate (TPR) was 21.9%, and about 80% of the land of the region was malarious, and 68% of the population was at risk of malaria in the study area from the data records of 1995 to 2020. The year 2012 to 2016 had the peak confirmed malaria cases, while the year 2016 to 2018 dramatically reduced followed by an increase in 2019/2020. The vignette FGDs identified that poor performance on Larval Source Management (LSM) and net utilization, no stock of some antimalarial medicine and supply, quality of malaria diagnosis services, the low commitment of leaders, and climatic anomalies facilitated surge of the disease in 2019/2020. No real accountability at all levels, low coverage of targeted vector control interventions, resource constraint, data quality and use for informed decision making, security issues and Internally Displaced Population (IDP) in various parts of the country, and the COVID-19 pandemic were the possible causes for case buildup.

**Conclusions:**

This result revealed that the malaria incidence rate showed a remarkable decline. However, the average TPR was 21.9%. Hence, it provided the ongoing feedback, mass fever test and treatment, training to health professionals, and ongoing supportive supervision (SS) and mentorship, improved net utilization and indoor residual spraying (IRS) operation and close follow-up and conducted sensitization workshop, spot messages were transferred through mass media, and temporary case treatment and prevention centers at farm sites established may surpass the threshold of malaria.

## 1. Background

Intestinal parasitic diseases such as malaria are curable. But, life-threatening infections cause intense febrile sickness, which taints the human liver and red blood cells. It is transmitted from individual to individual by the chomp of the *Anopheles* mosquito, with chomps as it were at night. It is transferred to individuals via the chomps of *Anopheles* mosquitoes, called malaria vectors, taints with *Plasmodium* parasites (PPs) [1].

In 2018, assessed 228 million diseases of malaria occurred globally (95% confidence interval (CI): 206–258 million), in contrast with 251 million infections in 2010 (95% CI: 231–278 million) and 231 million infections in 2017 (95% CI: 211–259 million). In the 2018, the World Health Organization (WHO) malaria report showed that the African region was with 213 million or 93%, followed by the Southeast Asia region with 3.4% of the diseases and the Eastern Mediterranean region with 2.1%, and in 2018, there were an estimated 405000 mortalities from malaria globally, compared with 416 000 determining mortality in 2017 and 585 000 in 2010 [2]. In low-income countries, malaria is very widespread, particularly in African countries including Ethiopia, causing high public health problems [3].

Trend analysis and exploring possible factors are vital for the well-timed distribution of information for action, and program evaluation results in the decrease of the cases through which time and place would be sustained. It clearly showed the continuing watch over the status of malaria in a community for detecting the changes in trends or distribution of malaria and other vector-borne diseases to initiate investigation or control measures and also strengthen the prevention and control of malaria in the community [4].

According to Ethiopia, malaria elimination strategy, mortality, and morbidity attributed to malaria declined significantly. Accordingly, death due to malaria declined by 67% from 0.9/100,000 population to 0.3/100,000 population at risk between 2016 and 2019. Similarly, the annual parasite incidence (API) has declined by 37% from the 19/1000 population to the 12/1000 population between 2016 and 2019. The number of confirmed malaria cases has reduced by 47% between 2016 and 2019. But, the decline was not sustained [5].

Trend analysis of infectious diseases is the keystone of Public Health (PH) decision making and practice, and also, exploring the possible factors which influence the malaria trend is crucial for malaria elimination programs [4].

The WHO recommended exploring the possible factors that positively and negatively influence the trend of malaria as a potential tool to reduce malaria transmission to reach malaria vectors that feed on the temporal and spatial gaps left by core vector control interventions [6].

Long year's trend analysis and exploring the factors also used for immediate PH action, program planning and evaluation, and formulating research suggestions were carried out. However, despite some years of trend analysis and some cross-sectional studies, a wide range of malaria trend needs and associated factors based on the existing variety of data sources essential to PH action in Ethiopia including the study site were not addressed well [7].

Therefore, the purpose of this study was to evaluate the trend in malaria cases and test the positivity rate and explore determinant factors in the Amhara Regional State, Ethiopia.

## 2. Methods

### 2.1. Study Design

#### 2.1.1. Quantitative Study

A retrospective follow-up study using health data records from 1995 to 2020 was employed.

#### 2.1.2. Qualitative Study

A case study, as a qualitative strategic design, was considered. Thus, the study employed a mixed-method approach.

### 2.2. Study Area and Period

The study was carried out in the Amhara Regional State, Ethiopia, from April 1, 2021, to June 30, 2021 G.C.

### 2.3. Study Unit

#### 2.3.1. Quantitative Study

All the malaria data registered during the study period in the study area were used.

#### 2.3.2. Qualitative Study

The study population was all malaria officers in the 12 zones of the region.

### 2.4. Sample Size and Technique

#### 2.4.1. Quantitative Study

The total malaria data were included in the study which were recorded from 1995 to 2020 considering the contextual manageability of things at the study area meaning to obtain meaningful regional and temporal malaria distribution during the year preceding the study by using a Purposive sampling system.

#### 2.4.2. Qualitative Study

A purposive sampling system was used to select those with in-depth information about the subject matter under study and collected the data using the Vignette Focus Group Discussions (FGDs). Eight vignette FGDs were conducted among 67 malaria officers (Oromia = 5, North Wollo = 5, North Shewa = 7, Waghimra = 5, South Wollo = 5, Awi = 5, West Gojjam = 8, East Gojjam = 7, North Gondar = 10, Gondar city = 1, Bahir Dar city = 2, and South Gondar = 6) considering data redundancy as a cut point for information saturation.

## 3. Data Collection Process

### 3.1. Development of the Data Collection Tool

#### 3.1.1. Quantitative Study

The data extraction tool was developed after reviewing varieties of related works of literature within the frame of the standardized malaria trend analysis checklist. Variables were coded and recoded, categorized, and recategorized by using the category of variables from credible sources such as regional health data sources.

#### 3.1.2. Qualitative Study

The data collection tool was developed in English after reviewing varieties of related works of literature and translated to the Amharic language (local language). Back translation to English was carried out by another person to check the consistency of words and notions.

### 3.2. Data Collection Procedure

#### 3.2.1. Quantitative Study

The information related to Malaria cases was retrieved from the Regional Data Health records using an information extraction sheet from April 2021 to June 2020 G.C by well-trained 5 BSc-holding nurses selected based on their data collection experience during their routine work supported by a Master of Public Health-holding immediate supervisor; then, the data were collected. Distantly was the principal investigator, who controlled the overall data collection process.

#### 3.2.2. Qualitative Study

Vignette focus group discussions were considered to elicit information on the reasons behind regional and temporal variation of malaria. All the data were recorded using a digital voice recorder, and notes were taken. A senior experienced journalist from the woreda government communication affairs office, in this case, not only assumed the ordinary position of a data collector, rather a critical interpreter of verbal and emotional talks of the study subjects, but also collected the data.

### 3.3. Data Quality Assurance

We reviewed the data by PH experts who have worked in the PHS system. Some data were deduplicated, especially the data from malaria monitoring charts, weekly PHEM reports, and year reports. One-day refreshment was given for data collectors and a supervisor on the overall data collection procedure to minimize systematic error, and also, on-spot checking and correction were made for incomplete case notes and checklists by the supervisor. Qualitative techniques that can increase the trustworthiness of this study (i.e., data and methodological triangulation, involving external agents in the course of data collection and analysis, and presentation of preliminary/pilot findings to colleagues**)** were employed.

### 3.4. Data Processing and Analysis

#### 3.4.1. Quantitative Data

The analysis in this study was based on retrospective malaria cases. So, we coded and entered the data using Excel 2016 and then exported it to RStudio-1.2.5033. The trend was figured as the regional and temporal variations. For all cases, the analysis and statistical tabular and graphical output was generated using RStudio-1.2.5033 for all years.

#### 3.4.2. Qualitative Data

The qualitative data generated from the focus group discussions within the group of Zonal Malaria Officers during the study period followed the principles of thematic content analysis. After listening to audio records several times, transcription of every word of the participants was considered accompanied by labeling and archiving of the narration into a pile of the labor arching file system. Thus, data were organized by questions to look across all respondents and their answers to identify consistencies and differences. Manual coding of the copied content of transcripts, then, was carried out to identify factors affecting the regional and temporal variation of malaria. Two higher-education instructors from Debre Tabor University coded selected transcripts in the first instance and compared them for consistency.

Then, discrepancies were appreciated after the investigator appraised the coding and emerged category results, and the remaining transcripts were divided among the coders, and coding was completed independently. Grouping of codes into mutually exclusive and exhaustive categories, as possible, to identify and build common themes across the dataset was also considered. To have a look over the relative importance of such categories, counting of the frequencies the theme comes up taken into account.

Furthermore, the formation of supercategories by combining some other categories was also considered to see how the parts relate to the converging single theme. Then, a short description was made for each category with quotes, as needed, from the text that illustrated the meaning. Eventually, cutting and sorting of the prelabeled data into the corresponding predefined categories were carried out followed by attachment of meaning and significance undertaken. Translation into English was carried out at a later stage after identifying meaningful themes.

## 4. Results

### 4.1. Trends of Confirmed Cases, Clinically Diagnosed Cases, and Positivity Rate of Malaria in the Amhara Regional State

According to the open-source statistical program R output, the average completeness of the weekly report collected from 1995–2020 G.C was 97.9% (95% CI: 96.2%, 98.9%). The average report timeliness was 96% (95% CI: 95.5%, 97.7%).

Based on their complete and timely report, we evaluated 1995 to 2020 confirmed, clinically diagnosed cases, total cases, the proportion of P.F, and the positivity rate of malaria in the region. The lowest positivity rate was observed in the year 1999/96/97/98 and 2017/18, while 1995/96/97 and 2010 had the highest positivity rate (Table 1).

**Figure 1:**
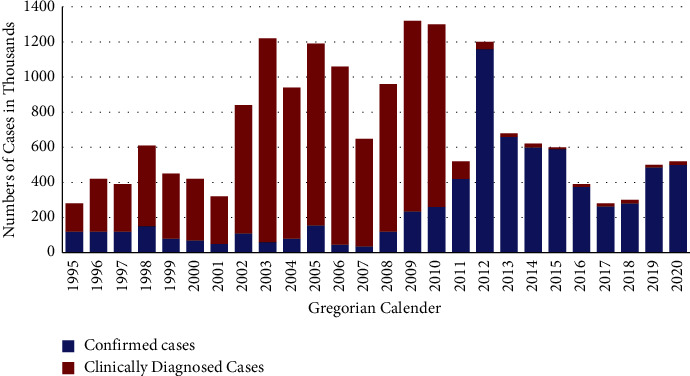
The open-source statistical program R graphical output of trend in confirmed and clinical malaria cases in the Amhara Region from 1995–2020.

In the current study, the clinically diagnosed cases were relatively high between 2002 to 2010 while confirmed cases were relatively high from 2011 to 2020 (Figure 1).

**Figure 2:**
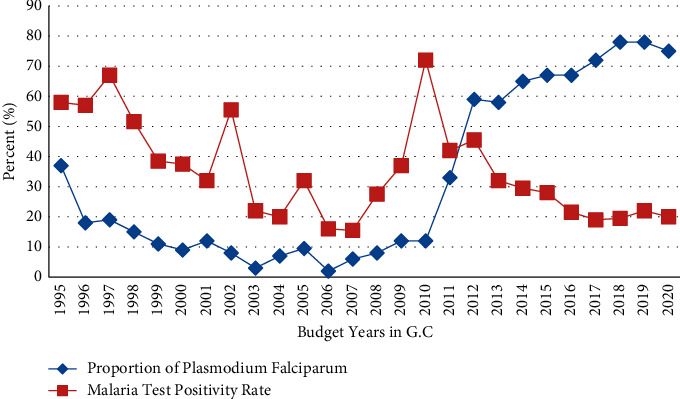
The open-source statistical program R graphical outputs of trend in malaria test positivity rate and PF proportion, Amhara Region, Ethiopia, from 1995 to 2020.

A total of 2,506,314 malaria suspected cases were tested (1,703,267 (68%) microscopic, 803,105 (32%) were using RDT with Overall Total Positivity Rate (TPR) = 21.9%, and P. F accounted for 75.2% of the confirmed malaria cases). The TPR reduced from 2010 to 2020, while P. F inversely increased (Figure 2 and Table 1).

**Figure 3:**
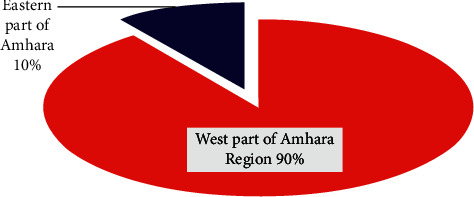
The regional malaria profile from 1995 to 2020.

About 80% of the land of the region was malarious, 68% of the population was at risk of malaria, 97% (163) districts reported cases, 83.7% (3,213) were malarious kebeles, and 9 districts (development corridors serve as sources for malaria transmission in the region (accounted for 30–35%/year) were among the 12 districts (Figure 3).

### 4.2. Determinant Factors of the Regional and Temporal Variation of Malaria

Most vignette FGD participants state that interruption of antimalarial commodities, low commitment of leaders, no real accountability at all levels, and low coverage of targeted vector control interventions due to the COVID-19 pandemic were found to be viewed as one of the major factors that made malaria cases increase by 36,004(7%) between 2017 and 2020.

Most of the participants from the vignette FGD claim climate change attributed to the possible causes for case buildup with the double burden of the COVID-19 pandemic, those climatic anomalies, which facilitated the surge of the disease in 2019/2020.

The vignette FGDs also reflected that the extent to which in the previous 5 years, most people are informed about the advantage of proper bed net utilization. Almost all necessary information about bed net use is available in the community. Information such as how to tuck the net under the bed, how to fumigate the home with smoke when the net is perceived to be nonfunctional, the irritation impact of the net on child skin, and how to wash and dry the net (though some of the procedures are wrong) are easily available resulting in the dramatic reduction of malaria from 2012 to 2018.

“Missed perception on the benefits of bed net such as carelessness and poor perception regarding bed net use is the problem, and poor implementation of rules and regulations is also a problem. Repurposing of the bed net is the most common practice in the community (especially in rural communities). Bed nets are used commonly for rope making, to harvest and cover the crop, to carry straw, and to kill insects such as bed bugs (laying under the matter to kill bed bugs and fleas with the chemical), and others highly increased during the current time.

Alongside this, not a few participants repeatedly asserted the poor performance on larval source management (LSM) and net utilization. Findings from almost all vignette FGD, equally, suggested the influence of immediate agents such as HEWs and other public wing agents (1 to 5 network and development group leaders) towards healthcare activities was dramatically reduced in 2019/2020 due to lack of financial support from the government and funding organization. In addition, resource constraints, data quality and use for informed decision making, no stock of some antimalarial medicine and supply, quality of malaria diagnosis services, climatic anomalies, which facilitated surge of the disease in 2019/2020, security issues and IDP in various parts of the country, and the COVID-19 pandemic were the main challenges which influence the malaria elimination and even control.

Objectionable sentimentalities of the participants are also evident. As per their quotes, in the previous year (before 2018), school malaria programs such as established antimalarial clubs in schools fulfilled minimedia materials and schools identified and posted permanent and temporary breeding sites, received/collected monthly reports from the woreda, and reviewed school performance at the regional level. Besides, vector control such as orientation of LLIN distribution campaign at all levels was given on time, regional close follow-up and monitoring of LLIN distribution were carried out by assigning individuals to specific woredas, daily LLIN distribution update was reported to the concerned bodies like a COVI19-19 report, weekly feedback was given to districts which surpass the threshold, training was given to health professionals, supportive supervision (SS) and mentorship has been conducted, LLINs were distributed and IRS operation was carried out, sensitization workshops were conducted, and spot messages were transferred through mass media which were comparatively good. Surprisingly, in our area, three interventions, case management (diagnosis and treatment), ITNs, and IRS, have given more emphasis rather than environmental management.

These major tasks were accomplished to contain case buildup which was more between 1999 and 2007; as a result, the malaria positivity rate reduced from 38.5% to 15.5%. So, we advised the government including ours and the partners to implement the tasks by identifying the malaria burden corridors.

## 5. Discussion

This study intended to evaluate the trend of malaria cases and positivity rate and its determinants from 1995 to 2020 G.C in the Amhara Region, Ethiopia, by describing and measuring indicators.

The overall report completeness and timeliness rate are above the World Health Organization minimum goals (80%) [4]. The difference might be due to increasing awareness of the community and acceptance of the community, health extension workers, and other health providers of the PHS.

The P.F prevalence rate of the current study is relatively matching with the national standard of the distribution of P.F, and the P.F prevalence increased by 75% in 2015 to 86% in 2016 [8]. However, it disagrees with the study conducted at the Butajira area, which dedicated an increased proportion of *vivax* malaria at high altitudes, the decrease in *vivax* malaria in the highland-fringe area, and the high transmissibility of *P. vivax* [9].

The mean TPR of this study is higher than that in the study conducted in Libokemkem District, Northwest Ethiopia. The difference might be that this study covers a large area, and the current study details the previous malaria status of the region [10].

Malaria is still a public health problem in the region irrespective of the past and existing vector control interventions. This is in line with the study conducted in Dembiya district, Northwestern Ethiopia [11].

The yearly anticipated occurrence of definite malaria is higher than twenty per thousand humans in the malarious and highly risky area of the region; this may be due to the higher number of reporting sites and arid areas and the presence of agricultural programs that could be affecting disease spread where incidence was higher than expected and the rest of the woreda was constantly under 5 reported malaria cases per thousand population per year [12]. In general, the use of prevalence while contrasting between woredas makes the zone more capable to map property properly, develop targeted diseases, organize labor, and permit an improved assessment of the program.

The vignette FGDs identified that poor performance on larval source management (LSM) and net utilization, no stock of some antimalarial medicine and supply, quality of malaria diagnosis services, a low commitment of leaders, climatic anomalies which facilitated surge of the disease in 2019/2020, no real accountability at all levels, low coverage of targeted vector control interventions, resource constraint, data quality and use for informed decision making, security issues and IDP in various parts of the country, and the COVID-19 pandemic were the possible causes for case buildup.

This is documented in the Ethiopian Public Health Institute report, 2012; there have been no well-organized epidemic preparedness and response planning and no financial and/or support. This could cause weak case detection and response during epidemics. Preparedness aims to strengthen capacity in recognizing and responding to public health emergencies through conducting regular risk identification and analysis, establishing partnerships and relationships, improving community participation, and implementing community-based interventions and strategic communication during the preemergency phase and ensuring their monitoring and evaluation [13].

The study also revealed that case management (diagnosis and treatment), ITNs, and IRS were one of the most important malaria prevention methods practiced in the locality. This is documented in the study conducted in the Chagni Health Center, Northwest Ethiopia [14].

### 5.1. Limitation of the Study

The study used a small sample size and was regional due to resource shortage.

## 6. Conclusions

This result revealed that the malaria incidence rate showed a remarkable decline and reverse. However, the average TPR was 21.9%. The study identified that poor performance on Larval Source Management (LSM) and net utilization, no stock of some antimalarial medicine and supply, quality of malaria diagnosis services, a low commitment of leaders, climatic anomalies which facilitated surge of the disease in 2019/2020, no real accountability at all levels, low coverage of targeted vector control interventions, resource constraint, data quality and use for informed decision making, security issues and Internally Displaced Population (IDP) in various parts of the country, and the COVID-19 pandemic were the possible causes for case buildup.

Hence, the weekly feedback was provided to districts, mass fever test and treatment were conducted, training was given to health professionals, ongoing supportive supervision (SS) and mentorship was conducted, improved net utilization and indoor residual spraying (IRS), operation based on area, and dose and close follow-up were carried out, sensitization workshops were conducted, spot messages were transferred through mass media, and temporary case treatment and prevention centers were established at farm sites that may surpass the threshold.

## Figures and Tables

**Table 1:** The open-source statistical program R outputs of the confirmed cases, clinically diagnosed cases, total cases, proportion of P.F, and positivity rate of malaria in the Amhara Regional State, Ethiopia, from 1995 to 2020.

Years	Confirmed cases	Clinically diagnosed cases	Total cases	Proportion of P.F	Positivity rate

1995	119	161	280	37	58
1996	120	300	400	18	57
1997	123	267	390	19	67
1998	150	460	610	15	51.6
1999	80	370	450	11	38.5
2000	70	350	420	9	37.5
2001	50	270	310	12	32
2002	110	730	840	8	55.5
2003	60	1160	1220	3	22
2004	80	860	940	7	20
2005	155	1035	1190	9.5	32
2006	45	1015	1060	2	16
2007	35	612	647	6	15.5
2008	120	840	985	8	27.5
2009	235	1085	1320	12	37
2010	260	1040	1300	12	72
2011	420	100	520	33	42
2012	1160	40	1200	59	45.5
2013	660	20	680	58	32
2014	600	20	620	65	29.5
2015	590	10	600	67	28
2016	375	15	390	67	21.5
2017	264	16	280	72	19
2018	280	20	300	78	19.5
2019	485	15	500	78	22
2020	500	20	520	75	20

## Data Availability

All the required data will be made available upon request to the corresponding author.

## Abbreviations and Acronyms

PHS: Public health surveillance
PH: Public health
P.F: *Plasmodium falciparum*
TPR: Total positivity rate.
